# Growth promotion and colonization of switchgrass (*Panicum virgatum*) cv. Alamo by bacterial endophyte *Burkholderia phytofirmans* strain PsJN

**DOI:** 10.1186/1754-6834-5-37

**Published:** 2012-05-30

**Authors:** Seonhwa Kim, Scott Lowman, Guichuan Hou, Jerzy Nowak, Barry Flinn, Chuansheng Mei

**Affiliations:** 1 Institute for Sustainable and Renewable Resource, Institute for Advanced Learning and Research, 150 Slayton Ave, Danville, VA, 24540, USA; 2 Department of Horticulture, Virginia Polytechnic Institute and State University, Blacksburg, VA, 24601, USA; 3 Department of Forest Resources and Environmental Conservation, Virginia Polytechnic Institute and State University, Blacksburg, VA, 24601, USA; 4 The Dewel Microscopy Facility at the College of Arts and Sciences, Appalachian State University, Boone, NC, 28608, USA

**Keywords:** Bacterial endophyte, *Burkholderia phytofirmans* strain PsJN, Colonization, Growth promotion, Biomass increase, Switchgrass cv. Alamo

## Abstract

**Background:**

Switchgrass is one of the most promising bioenergy crop candidates for the US. It gives relatively high biomass yield and can grow on marginal lands. However, its yields vary from year to year and from location to location. Thus it is imperative to develop a low input and sustainable switchgrass feedstock production system. One of the most feasible ways to increase biomass yields is to harness benefits of microbial endophytes.

**Results:**

We demonstrate that one of the most studied plant growth promoting bacterial endophytes, *Burkholderia phytofirmans* strain PsJN, is able to colonize and significantly promote growth of switchgrass cv. Alamo under *in vitro*, growth chamber, and greenhouse conditions. In several *in vitro* experiments, the average fresh weight of PsJN-inoculated plants was approximately 50% higher than non-inoculated plants. When one-month-old seedlings were grown in a growth chamber for 30 days, the PsJN-inoculated Alamo plants had significantly higher shoot and root biomass compared to controls. Biomass yield (dry weight) averaged from five experiments was 54.1% higher in the inoculated treatment compared to non-inoculated control. Similar results were obtained in greenhouse experiments with transplants grown in 4-gallon pots for two months. The inoculated plants exhibited more early tillers and persistent growth vigor with 48.6% higher biomass than controls. We also found that PsJN could significantly promote growth of switchgrass cv. Alamo under sub-optimal conditions. However, PsJN-mediated growth promotion in switchgrass is genotype specific.

**Conclusions:**

Our results show *B. phytofirmans* strain PsJN significantly promotes growth of switchgrass cv. Alamo under different conditions, especially in the early growth stages leading to enhanced production of tillers. This phenomenon may benefit switchgrass establishment in the first year. Moreover, PsJN significantly stimulated growth of switchgrass cv. Alamo under sub-optimal conditions, indicating that the use of the beneficial bacterial endophytes may boost switchgrass growth on marginal lands and significantly contribute to the development of a low input and sustainable feedstock production system.

## Background

Increasing concern over foreign energy supplies, global greenhouse gas emissions and the need for rural economic development has driven the interest in sustainable biomass production for bioenergy and bio-products. It has been suggested that by 2025, the world energy demand will likely be increased by more than 50% [1,2]. This demand, and societal concerns about the environmental impact of burning fossil fuels are key factors stimulating the development of national and regional strategies aimed at the growth of renewable energy supplies, primarily focused on biofuels. To reduce the reliance on fossil fuels, the USA, the world’s major energy consumer, released the Energy Independence and Security Act of 2007 that aims to increase the production of renewable fuels from 9.0 billion gallons in 2008 to 36 billion gallons by 2022 [3]. The recent USDA/DOE National Biofuels Action Plan [4] has helped to delineate the priority areas required to accelerate sustainable biofuel industry development. Within this document, Action Area 2 was identified as feedstock production and improvement. Various feedstocks, such as perennial rhizomatous grasses, can provide sources of lignocellulosic biomass, serving as new sources of crop growth and income for regional farmers.

One of the most promising feedstocks capable of contributing to the realization of US renewable energy goals is the common perennial grass, switchgrass (*Panicum virgatum* L.)[5]. This native prairie grass, consisting of a diverse germplasm [6], can grow on marginal lands under low inputs of water and agrochemicals [7], so that its cultivation does not compete with food crops for land and other resources. Due to its large root system and fast stand regrowth, switchgrass has other positive environmental effects, including the prevention of surface runoff and soil erosion, carbon sequestration, and the provision of a wildlife habitat [5,8]. Switchgrass cultivated lands also had much higher total soil organic carbon deposits than lands cultivated with annual crops, such as corn and wheat [9,10].

The economics of biofuel production is highly dependent on feedstock cost and conversion technology [1,6]. The development of improved switchgrass varieties for low-cost production on marginal lands is one prerequisite for the success of the bioenergy program [5,11]. One such approach involves the use of beneficial microorganisms, such as endophytes, which form intimate associations with plants [12,13]. Endophytes, both fungal and bacterial, have been targeted as mechanisms to enhance plant characteristics for commercial uses [14]. The colonization of grasses by fungal endophytes for performance enhancement is well documented [15], including their use with switchgrass [16,17]. However, to our knowledge, only one study has reported growth promotion of a bioenergy feedstock grass (*Miscanthus* x *giganteous*) seedlings by a bacterial endophyte (*Herbaspirillum frisingense*) [18]. A key component of our bioenergy crop research program involves the utilization of beneficial bacterial endophytes that form stable and persistent associations with switchgrass, as the mechanism to improve biomass yield and enhance stress tolerance under low-input production systems [19]. Beneficial bacterial endophytes are naturally occurring soil microorganisms that can penetrate plant roots and translocate to the above ground organs and, upon colonization, affect plant growth, health, and productivity [12,20-22]. Although the molecular mechanisms of beneficial endophyte-host plant interactions are largely unknown, several studies have demonstrated that endophytes can promote plant growth by enhancing the plant’s capacity for nutrient acquisition, better water management, and/or resistance to abiotic and biotic stresses via regulation of hormones [12-14,20,21]. For instance, 1-aminocyclopropane-1-carboxylic acid (ACC) deaminase produced by endophytes lowers the ethylene levels in host plants, reducing their response to abiotic and biotic stress, and by changing root morphology, leading to stimulation of plant growth [13,23,24]. Many known endophytes also promote plant growth by producing gibberellic acid (GA_3_)_,_ indole-3-acetic acid (IAA) [18,25], or cytokinins [26,27].

*Burkholderia phytofirmans* strain PsJN has been found to be a highly effective plant growth promoting bacterial endophyte, with a broad host range including potatoes, tomatoes, and grape vines [21,27-32]. In addition, its genome has recently been sequenced [33], providing the genomic resources needed to develop an understanding of the mechanisms associated with this endophyte’s ability to promote plant growth. PsJN produces a high level of ACC deaminase [31], enhances host plant cold [34] and heat [35] stress tolerance, improves water management [36] and plant resistance to pathogens [37,38]. In this study, we report growth promotion of switchgrass cv. Alamo by *Burkholderia phytofirmans* strain PsJN under *in vitro*, growth chamber, and greenhouse conditions. To our knowledge, this is the first report detailing the switchgrass-PsJN interaction.

## Results

### PsJN endophytic association with switchgrass Alamo

**Figure 1:**
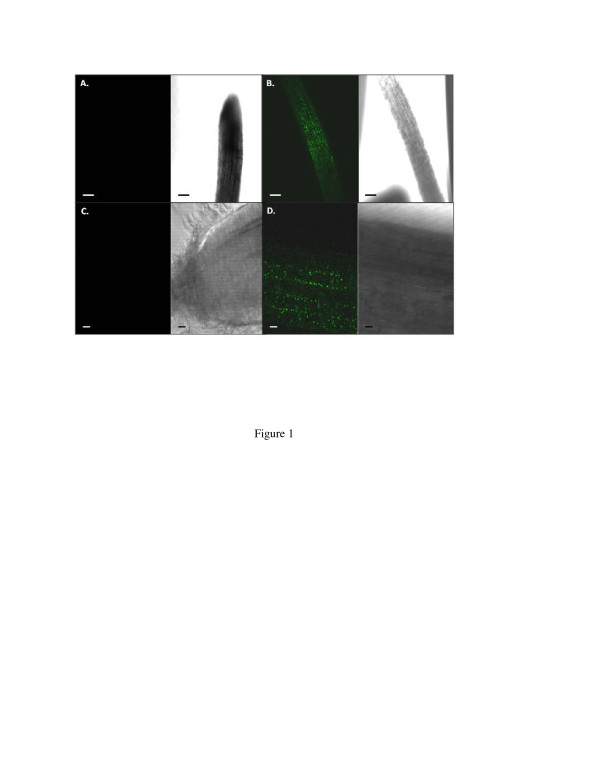
**Confocal images of roots taken 3 days following switchgrass cv. Alamo inoculation with PsJN-GFP, showing bacterial colonization inside the roots.(A)**: Control and **(B)**: PsJN- inoculated plants. **(C)**: Control and **(D)**: PsJN-inoculated plants. In each section (**a**,**b**,**c**,**d**), left panels were observed under fluorescent light and right panels under visible light. The bars represent 100 μm (A and B) and 20 μm (C and D).

The endophytic colonization of switchgrass by *Burkholderia phytofirmans* strain PsJN-GFP was visualized using confocal microscopy (Figure 1). Under the appropriate illumination, the PsJN-GFP could be clearly observed inside the roots of PsJN-inoculated plants 3 days after inoculation, while no fluorescence was observed in roots of buffer-inoculated control plants. The titer of PsJN-GFP in inoculated plants was also determined using tissue homogenates from various tissues (root, leaf and sheath) at different times (Table 1). The endophyte initially infected and colonized plant roots, and by 7 days post-inoculation, PsJN titers were still highest in the root. However, the titers increased significantly in other tissues by day 14, indicating translocation to leaves and sheaths.

**Table 1:** Colony-forming units (CFU) of root, leaf, and sheath tissues at different times after PsJN-GFP inoculation

**Days after PsJN-**	**Plant tissues**	**Average CFU/g**
**GFP inoculation**		**Fresh weight**
3	All (Roots, leaves, and sheath)	4.2X10^5^
7	Roots	7.6X10^5^
	Leaves	2.6X10^3^
14	Roots	3X10^4^
	Sheaths	1.3X10^5^
	Leaves	1.2X10^5^

### Effects of PsJN on Alamo growth *in vitro*

**Figure 2:**
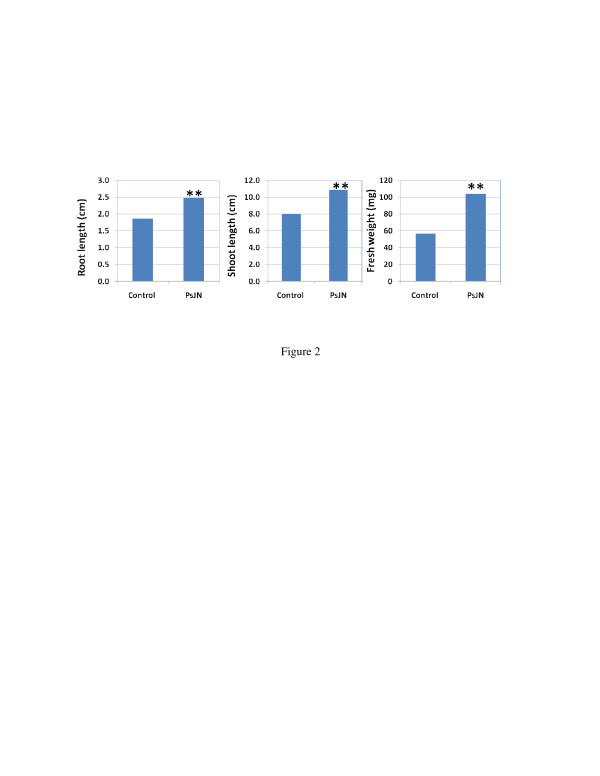
**Effects of endophyte PsJN inoculation on switchgrass cv. Alamo growth*in vitro***. Data were obtained after plants were grown in incubator for 36 days. Sample number was 25, and ** means significant difference at 0.01 level between PsJN and control using student *T*-test.

Young switchgrass seedlings were prepared and inoculated as described in Materials and Methods, and the non-inoculated and inoculated plants were analyzed after growth *in vitro* for one month. The result showed that PsJN significantly and repeatedly promoted Alamo root and shoot growth, with a 35.6% increase in shoot length, a 32.8% increase in root length, and an 83.6% increase in fresh weight, compared to the non-inoculated plants (Figure 2). After several replications, the average fresh weight of the PsJN-inoculated plants was always approximately 50% higher than non-inoculated plants.

### Effects of PsJN on Alamo growth in a growth chamber environment

**Figure 3:**
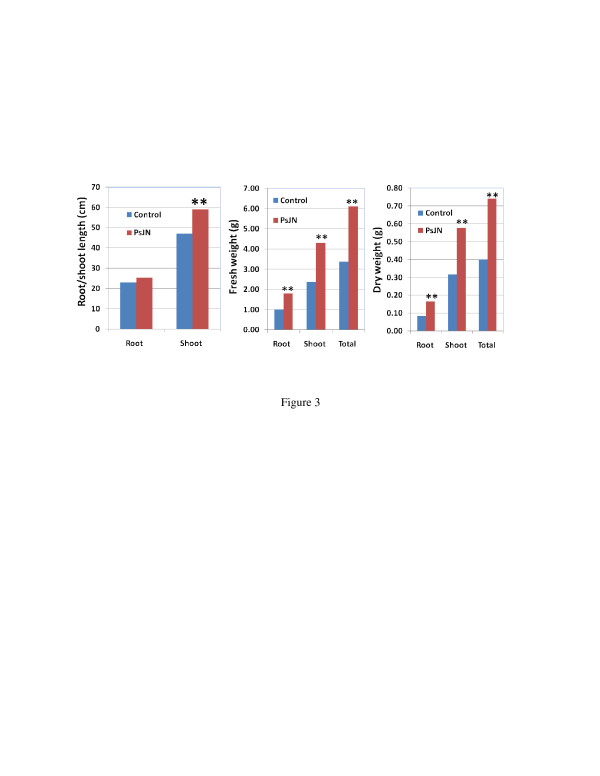
**Effects of endophyte PsJN inoculation on switchgrass cv. Alamo growth in growth chamber.** The seedlings were inoculated with PsJN and grown *in vitro* for one month, then transferred to soil and grown in growth chamber for another month. Dry weight was determined after samples were dried in oven at 65°C for one day. Sample number was 36, and ** means significant difference at 0.01 level between PsJN and control using student *T*-test.

As described above, PsJN significantly enhanced switchgrass cv. Alamo growth *in vitro*. We next assessed the impact of PsJN on growth under soil conditions. One-month-old *in vitro* grown Alamo (control and PsJN-inoculated seedlings) were transferred to a flat with 72 cavities filled with soil and grown in a growth chamber under 28/22°C day/night temperatures with 16 h light period for one month. The PsJN-inoculated plants showed significant growth increases compared to control plants in shoot length and fresh/dry weights (Figure 3). The growth chamber experiments were repeated 5 times, and the average data from 5 experiments showed significant growth promotion by PsJN, with a 46.3%, and a 54.1% increase in fresh weight and dry weight, respectively. The total dry weight increase (54.1%) by PsJN was more than the total fresh weight increase (46.3%), indicating that the PsJN-inoculated plants accumulated more biomass.

### Effects of PsJN on Alamo growth in the greenhouse

**Figure 4:**
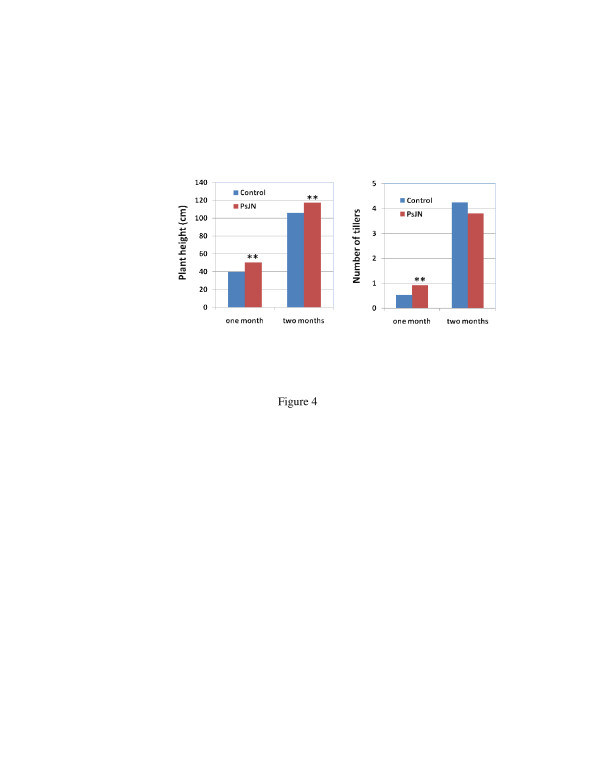
Plant growth and tiller development after control and PsJN inoculated plants were transferred to 4-gallon pots and grown in greenhouse.

**Figure 5:**
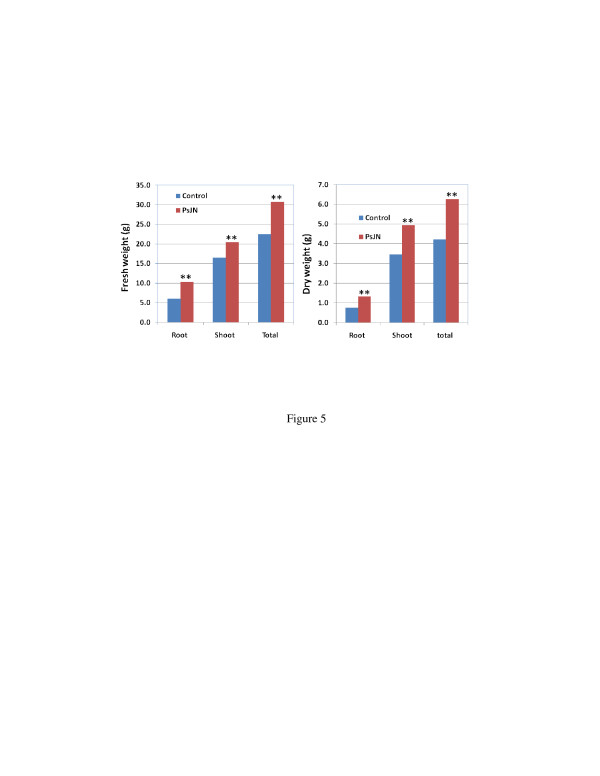
**Growth promotion persistence of Alamo in greenhouse by PsJN inoculation.** The seedlings were inoculated with PsJN and grown *in vitro* for one month, then transferred to 4-gallon pot with 5 plants/pot and grown in greenhouse for two months. Dry weight was determined after samples were dried in oven at 65°C for one day. Sample number was 25, and ** means significant difference at 0.01 level between PsJN and control using student *T*-test.

Non-inoculated and PsJN-inoculated plants were also grown under greenhouse conditions to determine growth enhancement persistence. The plants inoculated with PsJN and grown *in vitro* for 25 days were transferred to 4-gallon pots with 5 plants in each pot and grown in the greenhouse. The plants inoculated with PsJN exhibited sustained growth vigor, as they were significantly taller, and more tillers developed early compared with the non-inoculated control plants (Figure 4). Following one month of growth in the greenhouse, the PsJN-inoculated plants had 76.2% more tillers than the control plants. The plants were harvested following growth for two months, and the biomass yield determined (Figure 5). The PsJN-inoculated plants were repeatedly significantly higher in biomass yield, with a 36.8% increase in fresh weight and a 57.1% increase in dry weight.

### Effects of PsJN on Alamo growth in sub-optimal conditions

**Figure 6:**
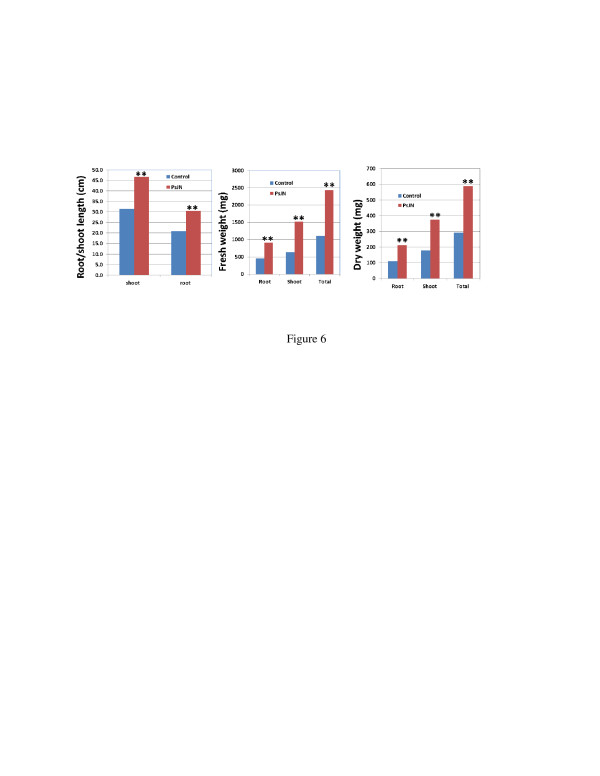
**Growth promotion of Alamo by PsJN inoculation in sub-optimal conditions.** The seedlings were inoculated with PsJN and grown *in vitro* for one month, then transferred to 4-gallon pots with 5 plants/pot with unfertilized field soil and grown in the glasshouse under ambient conditions for 2.5 months in the late Fall of 2010. Dry weight was determined after samples were dried in oven at 65°C for one day. Sample number was 25, and ** means significant difference at 0.01 level between PsJN and control using student *T*-test.

In order to develop a low input and sustainable switchgrass feedstock production system utilizing the beneficial bacterial endophyte, we tested growth performance of PsJN-inoculated plant with unfertilized field soil, in a glasshouse under ambient conditions during the Fall, when the temperature was not optimal for switchgrass growth. The results showed that PsJN-inoculated plants produced twice the total biomass of controls (Figure 6).

### Direct inoculation of switchgrass seeds with PsJN

**Figure 7:**
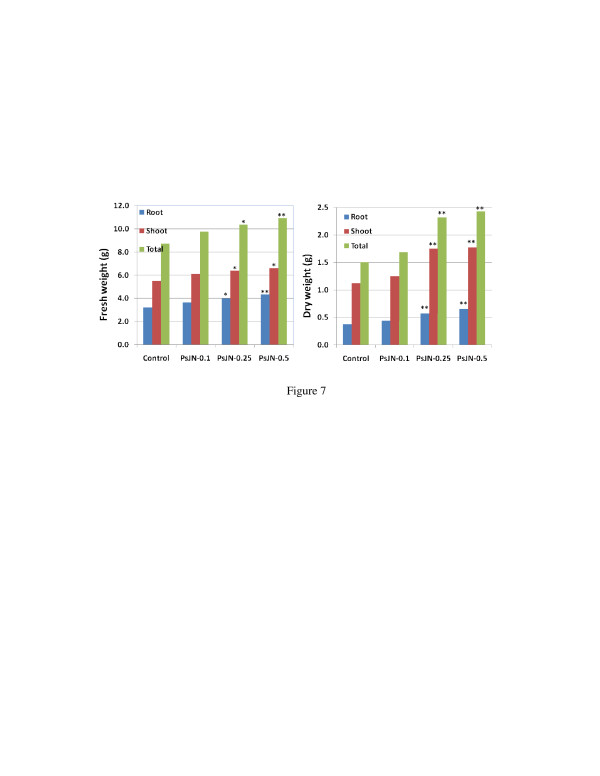
**Effects of different concentrations of PsJN on swtchgrass cv. Alamo growth with direct seed inoculation.** The surface-sterilized seeds were infected with different concentrations of PsJN and grown *in vitro* for 17 days, then transferred to 72-cavity trays and grown in a growth chamber for 50 days. * and ** mean significant difference at 0.05 and 0.01 levels respectively between PsJN and control using student *T*-test.

In order to explore a practical way to inoculate switchgrass with the bacterial endophyte, we sterilized switchgrass seeds as described in Materials and Methods, placed the sterilized seeds on wet filter paper for 3-5 days in an incubator at 25°C, and then inoculated the germinating seeds with different concentrations of endophyte inoculum to determine the optimal inoculation concentration (OD_600_ at 0.1-0.5). The plants inoculated with PsJN at OD_600_ of 0.1, 0.25, and 0.5 exhibited 28.7%, 55.0% and 80.1% increases in dry weight, respectively, compared to non-inoculated plants after grown *in vitro* for 25 days and in growth chamber for another month. A PsJN concentration of 0.5 was the most effective at promoting biomass increase (Figure 7), and no biomass difference was observed between the 0.1 treatment and control.

**Figure 8:**
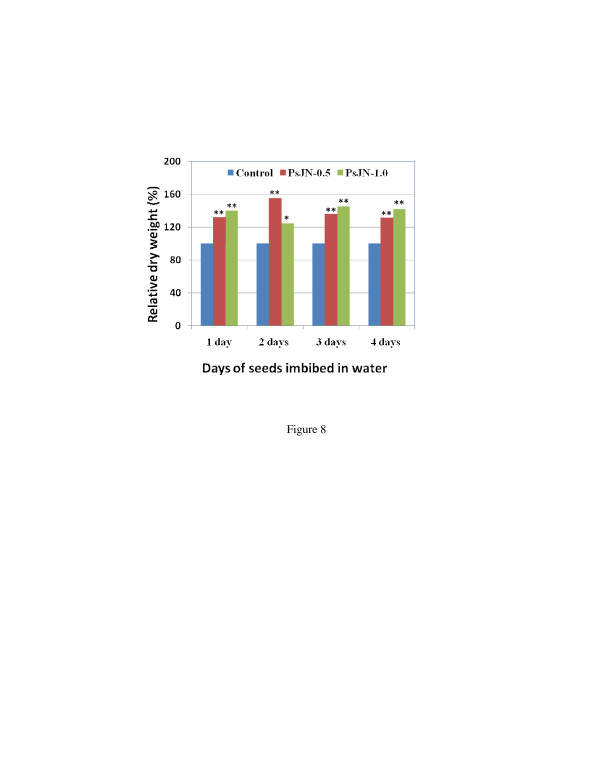
**Effects of different concentrations of PsJN on switchgrass cv. Alamo growth with direct inoculation of seeds that were imbibed in water for different days (1-4 days).** The seeds were infected with different concentrations of PsJN and grown *in vitro* for 25 days, then transferred to 72-cavity trays and grown in a growth chamber for 37 days. Sample number was 72 for each treatment. * and ** mean significant difference at 0.05 and 0.01 levels respectively between PsJN and control using student *T*-test.

Endophyte infection and colonization of seeds are dependent on endophyte concentration and the status of seed imbibitions. So, in order to facilitate infection and colonization by the bacterial endophyte, the sterilized seeds were imbibed in water for 1, 2, 3, or 4 days, and then inoculated with PsJN at an OD_600_ of 0.5 or 1.0, since an OD_600_ of 0.5 was the most effective as described above. The PsJN-inoculated seeds were placed in an incubator at 25°C with a 16 h light period for 25 days, and transferred to soil and grown in a growth chamber for 37 days. The results indicated that plants from the seeds imbibed for 2 days and then inoculated with an OD_600_ of 0.5 had the highest dry weight, with a 55% increase compared to non-inoculated control plants (Figure 8).

### Genotypic responses to PsJN

As described above, PsJN was able to stimulate growth in switchgrass cv. Alamo. To assess the influence of plant genotype on this response, seven other switchgrass cultivars were tested for their growth responses to PsJN. As shown in Table 2, growth promotion by PsJN was genotype-dependent. The switchgrass cvs. Forestburg, Nebraska, and Blackwell were all responsive to PsJN, with measured significant growth increases of 60.1%, 26.8%, and 23.0%, respectively, while the cvs. Cave-in-Rock, Sunburst, Shelton, and Shawnee did not exhibit growth promotion in response to PsJN under similar conditions. Preliminary result from Cave-in-Rock bioassay indicated that PsJN titers were not sustained after inoculation and were much lower in the non-responsive plants following inoculation.

**Table 2:** Effects of *B. phytofirmans* PsJN on plant growth in different switchgrass cultivars

**Cultivars**	**Treatment**	**No. plants**	**Root length (cm)**	**Shoot length (cm)**	**Total fresh weight (mg)**	**PsJN/control (%)**^**a**^
Shawnee	Control	24	2.3	8.4	50.1	
	PsJN	24	2.1	7.8	58.8	117.4
	p-value^b^		0.2037	0.2049	0.0717	
Nebraska	Control	24	1.5	10.3	40.9	
	PsJN	24	1.5	11.6	51.9	126.8
	p-value		0.4160	0.0656	0.0055	
Forestburg	Control	34	1.6	8.6	33.7	
	PsJN	30	1.8	12.7	54.0	160.1
	p-value		0.1986	0.0000002	0.0000001	
Shelton	Control	28	3.3	14.1	117.7	
	PsJN	28	3.0	12.0	135.3	115.0
	p-value		0.1579	0.0067	0.0907	
Blackwell	Control	28	1.4	9.9	52.6	
	PsJN	28	1.5	11.3	64.7	123.0
	p-value		0.2970	0.0998	0.0543	
Sunburst	Control	30	0.8	8.5	33.0	
	PsJN	28	0.9	10.4	26.4	80.0
	p-value		0.2985	0.0137	0.0731	
Cave-in-Rock	Control	33	2.8	14.7	107.8	
	PsJN	34	3.1	13.8	113.4	105.2
	p-value		0.2317	0.4052	0.5613	

^a^ PsJN/control (%) means total fresh weight of PsJN inoculated plants over that of control plants.

^b^ p-value means degrees of statistical significance between PsJN treatment and control using student *T*-test.

## Discussion

In the present study, we demonstrated the ability of *B. phytofirmans* PsJN to colonize and promote growth in switchgrass cv. Alamo. Three days following PsJN inoculation, we could clearly visualize bacterial colonization inside the roots under confocal microscopy. The bacterial population inside the roots was initially much higher than that of the leaves and sheaths, and the bacterial endophyte was subsequently translocated vertically to the upper leaves through the leaf sheath. These results were similar to that reported for grapevine [22] and potato [39], where PsJN was transported through the interior vascular system, from root xylem vessels to the upper parts of the plants. This is a critical first step in the endophytic bacteria-plant interaction [40]. We observed significant growth promotion of cv. Alamo by PsJN, under both *in vitro* and soil conditions. Our study showed total fresh weight and total dry weight of the inoculated plants was increased by 45% and 55% respectively compared with the non-inoculated control plants when the inoculated seedlings were grown *in vitro* and then transferred to soil and grown in growth chamber for one month. Similar results have been obtained in 4-gallon pots under our greenhouse conditions. Other studies have reported levels of growth promotion by PsJN, with grapevine showing a 6-fold increase in total biomass [34], and potato showing an approximate 2-fold increase in root and haulm biomass [36] over controls. The mechanism of plant growth by *B*. *phytofirmans* PsJN has been reported [21] and attributed to the ability of PsJN to produce high levels of ACC deaminase activity, which degrades ACC to ammonia and α-ketobutyrate [41], which is a common characteristic of plant growth promoting bacteria. ACC is the precursor to ethylene, a plant stress hormone; hence, the reduced ethylene level in PsJN-colonized plants will promote plant growth. According to the report by Penrose and Glick (2003) [42], ACC activity over 20 nmol α-ketobutyrate/h/mg is sufficient to promote host plant growth, and PsJN has been shown to contain 308 nmol α-ketobutyrate/h/mg of ACC deaminase activity [31]. Although several studies have reported the interaction between this endophyte and host plants for growth promotion, most studies have reported *in vitro* data [22,27,28,36]. Our results with unfertilized field soil, in a glasshouse under ambient conditions during the Fall, when the temperature was not optimal for switchgrass growth (Figure 6) implied the potential benefit of switchgrass inoculated with PsJN for growth on marginal lands and sub-optimal growth conditions. Although our results indicate potential benefit of bacterial endophytes in switchgrass under *in vitro*, growth chamber and greenhouse conditions, much work still needs to be done in assessing growth promotion of switchgrass by endophytes in the field since there is much more competition between endophytes and other microorganisms in addition to many other unfavorable conditions. While our initial studies were carried out with cv. Alamo, we tested the utility of PsJN as a growth-promoting endophyte with other switchgrass cultivars. Our results indicated that specific genotype effects existed, with some genotypes being highly responsive to the growth promotive effects of PsJN, and others not. Similar genotype effects have been reported by others. It was reported that the potato response to PsJN involves some form of genetic control, as some potato cultivars display the beneficial response to the endophyte, while others do not [35,43,44]. The typical *in vitro* phenotype for a strongly responsive cultivar was characterized by a massive, well-branched root system and after the first 3-4 weeks in culture, the plantlet was developmentally more advanced than the non-bacterized controls. Stems were sturdier, with more lignin deposits around the vascular system, and the plantlets developed more root hairs and more and larger leaf trichomes [43]. We also noticed PsJN-inoculated switchgrass plants were developmentally advanced (unpublished data). Such enhancements were not apparent for the poorly-responsive cultivars. We also observed some of these phenotypic differences between PsJN-responsive and non-responsive switchgrass cultivars. Additional work illustrating the genetic control of the beneficial response to the endophyte used monoploid potato lines derived from anther culture of an adapted diploid *Solanum phureja* clone, BARD 1-3 [44]. The diploid anther donor, BARD 1-3, exhibited a bacterization response comparable to Red Pontiac, while monoploid lines exhibited a response to PsJN ranging from favorable to unfavorable to neutral. The assumption here was that the response range of the monoploid population was due to the segregation of alleles for genes involved in regulating the positive or negative interaction with PsJN.

The potato/PsJN studies have been the most characterized, and carried out with material clonally propagated via nodal sections, in which a single inoculation is sufficient to initiate colonization through the xylem tissue, eventually spreading to the upper leaves [36]. Bacterial levels must reach a threshold population within the plant before they are effective [45] with a direct relationship between plantlet growth enhancement and PsJN colonization of both interior and exterior surfaces [44]. The PsJN colonization profiles for a responsive and poorly responsive cultivar over 8 tissue culture generations revealed bacterial loads one order of magnitude greater for shoot/root surface and interior colonization in the responsive compared to the poorly responsive cultivar. Furthermore, the responsive cultivar exhibited increased colonization over successive generations, while the poorly responsive cultivar exhibited declining bacterial populations over successive generations. We are currently assessing the level of colonization in switchgrass cultivars responsive and non-responsive to PsJN to determine the degree of similarity between switchgrass and potato responses to the endophyte. At present, the mechanisms governing *B. phytofirmans* PsJN genotype-specificity in growth promotion of switchgrass (and other plants) are unknown, although we are currently using large scale gene expression analyses to determine the differences in the switchgrass molecular responses between differently-responding cultivars. In summary, the results reported here illustrate the ability of *B. phytofirmans* PsJN to infect and colonize responsive switchgrass (*Panicum virgatum*) cultivars like Alamo, and to promote plant growth. This study lays the foundation to develop a low input and sustainable switchgrass feedstock production system on marginal lands using this, and other, beneficial bacterial endophytes.

## Conclusions

Results obtained with growth promotion of switchgrass cv. Alamo by *B. phytofirmans* PsJN under various growth conditions, sub-optimal in particular, indicate the potential for utilization of beneficial bacterial endophyte in switchgrass establishment in the first year and in the development of low input and sustainable switchgrass feedstock production system. In the future, the mechanisms of growth promotion need to be elucidated with molecular biology and functional genomics to develop tools for molecular breeding for beneficial plant-microbial associations.

## Methods

### Plant materials

Switchgrass (*Panicum virgatum* L.) cvs. Alamo and Cave-in-Rock seeds were purchased from Warner Brothers Seed Co. (Lawton, OK), and other switchgrass seeds were kindly provided by Dr. Bingyu Zhao (Department of Horticulture - Virginia Tech, Blacksburg, VA). Switchgrass seeds were surface-sterilized by treatment with 70% ethanol for 2 min, rinsed 3X with distilled water, de-husked for 30 min with 60% H_2_SO_4_ with stirring, washed 3X with distilled water, sterilized with 0.4 M sodium hypochlorite (50% commercial bleach solution containing 6% sodium hypochlorite) containing 0.1% Triton 100 for 30 min followed by 5X rinse with sterile, deionized, distilled water (ddH_2_O).

### Bacterial endophyte and culture conditions

*Burkholderia phytofirmans* strain PsJN [31] and its PsJN-GFP derivative [22] were obtained from Dr. Angela Sessitsch (Austrian Institute of Technology, Seibersdorf, Austria). The cultures were streaked on King’s B (KB) solid medium as described in [45]. Inoculum was produced by transferring one loop of PsJN from 2-day-old cultures to 5 ml KB broth in a 15-ml culture tube, followed by incubation at 28°C on a shaker (150 rpm) overnight. Five ml of the overnight PsJN culture was added to 45 ml KB broth in a 250-ml Erlenmeyer flask and grown to 0.7 OD_600_. Bacterial cells were then collected by centrifugation at 3,500 rpm for 7 min at 4°C, and re-suspended in PBS buffer (10 mM NaH_2_PO_4_ containing 0.8% NaC1, pH 6.5) after which the OD_600_ was adjusted with PBS buffer to 0.5, unless described otherwise.

### Seedling inoculation with PsJN and plant growth responses

Surface-sterilized seeds were germinated in petri-dishes for 7 days at 25°C, under white fluorescent light (67 μmol m^-2^ s^-1^), 16 h photoperiod, on a switchgrass growth medium consisting of MS salts and vitamins [46], 30 g/l maltose and 3 g/l phytogel, pH 5.8. The root tips of the young seedlings were cut prior to PsJN inoculation to facilitate bacterial penetration [45]. For the direct seed inoculation surface-sterilized seeds were placed on wet filter paper for 3-5 days in an incubator at 25°C with 16 h photoperiod (white fluorescent bulbs at 67 μmol m^-2^ s^-1^) followed by soaking in PsJN solution (0.5 of OD_600_) for 1 min. Control seedlings/seeds were treated with PBS buffer alone. The treated seedling/seeds were blot-dried with sterile paper towel, placed on switchgrass growth medium in GA7 Magenta vessels (Sigma-Aldrich) containing 50 ml of media and 5 seedlings or germinating seeds per vessel, and grown for one month in the incubator as above. Root and shoot length, and seedling fresh weight were then determined, and the plants transferred to a soil mix composed of 2/3 Miracle-Gro® Potting Mix (Scotts Miracle-Gro Company, Marysville, Ohio) and 1/3 Arabidopsis growing media (Lehle Seeds, Round Rock, Texas). Plants were grown in 72-cavity trays in a growth chamber at a 28/22°C day/night temperature, 16 h photoperiod (white fluorescent bulbs at 88 μmol m^-2^ s^-1^) for 30 days, or 4-gallon pots in the greenhouse.

### PsJN colonization

The plants inoculated with PsJN-GFP were examined under a fluorescent stereomicroscope (Model SZX-ILLD2-100; Olympus, Tokyo, Japan) equipped with a GFP filter (BP460-490, Olympus, Tokyo, Japan) and the Zeiss 510 laser scanning confocal microscope (LSCM) (Carl Zeiss, Inc., Thornwood, NY) to observe colonization.

For bioassays, the control and PsJN-GFP inoculated plants were surface-sterilized with 0.032 M sodium hypochlorite for 1 min, then washed 4X with sterile distilled water. Fifty μl of the final wash was plated on solid KB medium to confirm effectiveness of surface sterilization. Root, leaf and sheath parts were then separated, each weighed, and ground with mortar and pestle in 1 ml sterile distilled water. The homogenates were then centrifuged at 2000 rpm for 3 min, and the supernatant diluted to 1:10, 1:100, and 1:1000 with distilled water. Fifty μl samples of the serially diluted solutions were spread on solid KB medium. The plates were incubated for 3 days at 28°C in the dark and the number of GFP-positive colonies determined using fluorescence stereomicroscopy as described above.

## Competing interests

The authors declare that they have no competing interests.

## Authors’ contributions

SK and SL conducted experiments and drafted the manuscript. GH carried out confocal imaging and data analysis and reviewed the manuscript. CM, JN and BF designed experiments and wrote the paper with input from all authors. All authors read and approved the final manuscript.
